# Disentangling the dynamic interplay between muscle damage and energetics in male boxers during a short training block

**DOI:** 10.5114/biolsport.2024.127383

**Published:** 2023-05-30

**Authors:** Zbigniew Obmiński, Blair T Crewther, Christian J Cook

**Affiliations:** 1 Institute of Sport – National Research Institute, Warsaw, Poland; 2 Biomedical Discipline School of Science and Technology, University of New England, Armidale, Australia; 3 Hamlyn Centre, Imperial College, London, UK

**Keywords:** Recovery, Competition Combat, Sport, Injuries, Glomerular, Filtration

## Abstract

Boxing is a combat sport linked to muscle damage (e.g., soreness, rising creatine kinase [CK]) and energetic biomarkers (e.g., urea, glucose). These factors have not, however, been examined dynamically in terms of day-to-day, lagged and reciprocal effects during normal training. This study investigated the dynamic interplay between muscle damage and energetics in male boxers during a short training block. Thirteen amateur boxers were monitored over 16 consecutive days during early-season training. The participants were assessed each morning for plasma CK, urea, glucose, and creatinine (days 1 and 16 only) concentrations, before self-reporting muscle soreness (1–10 scale). Within-person contemporaneous (lag-0) and temporal (lag-1) networks were estimated using multilevel vector autoregression. Muscle soreness, CK, urea, and glucose presented different trajectories with training, but with some heterogeneity reflecting within-person variances (47% to 78%). The contemporaneous network yielded a significant positive edge (or correlation) between CK and soreness (r = 0.44), along with negative CK-glucose and glucose-urea edges. More significant edges emerged in the temporal network, with soreness linked to CK (r = 0.19), glucose (*r* = -0.28) and urea (*r* = 0.22), whilst the CK-glucose edge sign switched. In summary, daily fluctuations in muscle damage and energetic activity, which presented in a normal physiological range, were highly variable among boxers during early-season training. Within-person networks indicated some interrelatedness between CK, soreness, urea, and glucose, although the nature and presence of these relationships were contingent on temporal ordering. These inconsistences reflect the pleiotropy of energetic biomarkers in training and recovery.

## INTRODUCTION

Boxing is a combat sport characterized by short duration, high-intensity bursts of physical activity and impact [1, 2, 3]. Physiologically, boxing matches and sparring are associated with high-energy turnover, leading to activation of energetic biomarkers (e.g., glucose, cortisol, lactate) and micro-injuries that provoke an inflammatory response [2, 3, 4, 5]. Many factors can influence these parameters (at rest or after exercise) among boxers, like age and training status [1], whether the activity is traumatic (i.e., real boxing) or simply muscular work (i.e., shadow boxing) [6], dietary factors (e.g., protein supplementation) [7], and any post-exercise recovery strategies used [8]. Whilst this work is informative, little is known about the training response of boxers [9, 10]. In particular, how these factors interact on day-to-day basis, including lagged and reciprocal effects, in an ecological training environment.

This research sought to disentangle the daily interplay between muscle damage (i.e., muscle soreness, CK) and energetic activity (i.e., urea, glucose) in male boxers during a short training block. Measurements were collected over 16 consecutive days. We first examined the group and individual trajectories over time, before simultaneously modeling the entire dataset using multivariate network analyses. By taking a network approach, we view the muscle damage-recovery process as a “dynamical system” in which the factors described affect, and are affected by, each other on different time scales. Within-person networks were emphasized to accommodate individual-specific responses to exercise-induced muscle damage (EIMD) [4, 11, 12]. As an exploratory study, no firm hypotheses were generated.

## MATERIALS AND METHODS

### Participants

Thirteen amateur male boxers were recruited for this study, with a mean (± SD) age and body mass of 25.1 ± 1.5 years and 67.1 ± 13.2 kg, respectively. The athletes were classified as elite performers, who were competing at a national and/or international level, with an average training experience of 11.1 ± 1.5 years. The participants did not report taking any drugs, doping agents or special supplements at the time of this study (or prior to), nor did they report any injuries or medical problems that would affect the study results or their ability to train. Ethical approval was obtained from the Institute of Sport – National Research Institute, Poland, with full adherence to ethical standards in sport and exercise research [13]. For example, each athlete received a full briefing on the study aims, experimental procedures, and potential benefits, before providing written and verbal informed consent. Participation was completely voluntary, so they could leave the study at any time without prejudice.

### Study design

A longitudinal, observational study was undertaken involving the daily assessment of male boxers for muscle damage and energetic activity over 16 consecutive days. Data were collected across an early-season training block, which represented the first training camp in February (Polish winter months) following a 1-month detraining period. All athletes were housed together where they completed the same training regime, followed a similar sleep-wake schedule, and consumed a similar diet via a set daily menu. Individual dietary intake was not strictly monitored during this study, but we anticipated that typical macronutrient needs for athletes (e.g., 50–60% carbohydrates, 10–20% protein, 20–30% fat) would be naturally adhered to. We assumed high calorie intake by these athletes without energy restriction, as required for preparatory training, and adequate hydration throughout. The modality, intensity and format of training was the same for all athletes during this preparatory period; see below for more specific details.

Athlete training comprised of moderate- to high-intensity exercise that included a combination of; (1) general physical conditioning (e.g., shuttle runs, push-ups, pull-ups, skipping) in both interval and continuous formats, (2) heavy bag work in interval format to simulate a boxing contest (3–5 x 3-min rounds, 1-min recovery), (3) one-on-one boxing drills involving pad work with a trainer, (4) full-contact sparring (3 x 3-min rounds, 1-min recovery) with both participants wearing gloves and head protection. The sparring workouts were preceded by shadow boxing and some bag work, as a warm-up procedure. The boxers completed 1–2 training sessions a day, each lasting ~1 hour, with exercise intensity gradually increasing (i.e., greater work to recovery ratio) from day 1 up to day 16. Exercise intensity was subjectively assessed by the coach and training staff using visual / verbal cues of perceived effort, movement quality, and fatigue. The study participants were scheduled to train on Monday to Saturday of each week with Sundays free of any physical activity.

### Assessments

Capillary blood samples (~200 μL) were collected from the earlobe into heparinized tubes. The tubes were centrifuged and the plasma portion was extracted into another tube for storage in a -80° C freezer. Each sample was tested for creatinine (in μmol/L), CK (in U/L), urea (in mmol/L), and glucose (in mmol/L) concentrations using a biochemical analyzer (Biotecnica Instruments, Italy) and reagents supplied by the manufacturer. Note that plasma creatinine was assessed on days 1 and 16 only. Capillary-based measurements of CK, urea, and glucose [14, 15, 16] are reliable and also valid, compared with venous blood collections, although the measured concentrations are not always interchangeable between blood compartments.

General muscle soreness was assessed daily by self-report and anchored on a 1 (= no pain) to 10 (= extremely painful) Likert scale [12, 17]. These data were collected with a time-framed question (i.e., how sore are your major muscle groups right now?), after which the participant was shown a card with all possible ratings and explanations. Body mass was measured to the nearest 0.1 kg on days 1 and 16 using electronic scales (Seca, Germany), with subjects wearing training shorts and a shirt, but without shoes.

### Statistical analyses

Data were analyzed in the R programming environment [18]. First, descriptive means and SDs were calculated for all variables, based on averages of all within-person results. Intraclass correlation coefficients (ICC) were also calculated for selected variables (i.e., soreness, CK, urea, glucose) to extract the variance components. In other words, is the observed variability due to within-person (e.g., CK changes over time for all subjects) or between-person (e.g., CK differences among subjects) sources. Next, a visual inspection of the variable trajectories was conducted to identify group patterns and individual differences from the group mean. To do this, we plotted the smoothed means of the study population using a generalized additive model, overlaying all individual data points.

Two within-person networks were estimated using multilevel vector autoregression (VAR) in the mlVAR package [19]. The first is a *temporal network* reflecting within-person partial correlations of the average person at lag-1 (1-day), and second is a *contemporaneous network* reflecting within-person partial correlations of the average person at the same measurement occasion (lag-0). In networking parlance, correlations between variables are termed edges and variables are termed nodes [20]. Prior to analysis, checks for VAR assumptions of stationarity, multivariate normality, and linearity were performed. To meet these assumptions, the glucose data were regressed onto time and the residuals saved as the new variable. The study design ensured that the VAR assumption of equidistant intervals was met. In line with recent studies using different dynamical-system modeling approaches [21, 22, 23], all time-series data were standardized within a person prior to network estimation. The ensuing network edges were thresholded at *p* < 0.05. No edge adjustments were made for multiple comparisons, as we wanted to err on the side of discovery.

## RESULTS

The descriptive and stability results are presented in Table 1. Athlete ratings of muscle soreness, although stimulus and timing dependent, are similar to other EIMD studies on untrained adults [12, 17] and male boxers [7]. The concentration measures of plasma creatinine, CK, urea, and glucose all lie within a normal physiological range, and overlap reported values in other boxing studies [1, 2, 4, 5, 6, 7, 9, 10, 24]. All ICCs were small to moderate for muscle soreness and the plasma biomarkers, varying between 0.22 and 0.53, which means that 22% to 53% of the variances originated at the between-person level. Thus, the remaining variances (47% to 78%) can be attributed to within-person sources.

**TABLE 1:** Descriptive and reliability statistics for the muscle damage and energetic variables in male boxers across a short training block.

Variables	Mean	SD	ICC	Sample range (min – max)
Soreness (1–10)	4.17	1.37	0.22	1–9
Creatinine (μmol/L)*	84.7	3.64	na	54–118
CK (U/L)	650	297	0.52	88–2573
Urea (mmol/L)	6.72	0.82	0.53	4.1–10.0
Glucose (mmol/L)	4.59	0.28	0.25	4.0–5.6

Key: CK = creatine kinase.

* Assessed on days 1 and 16 only.

**FIG. 1:**
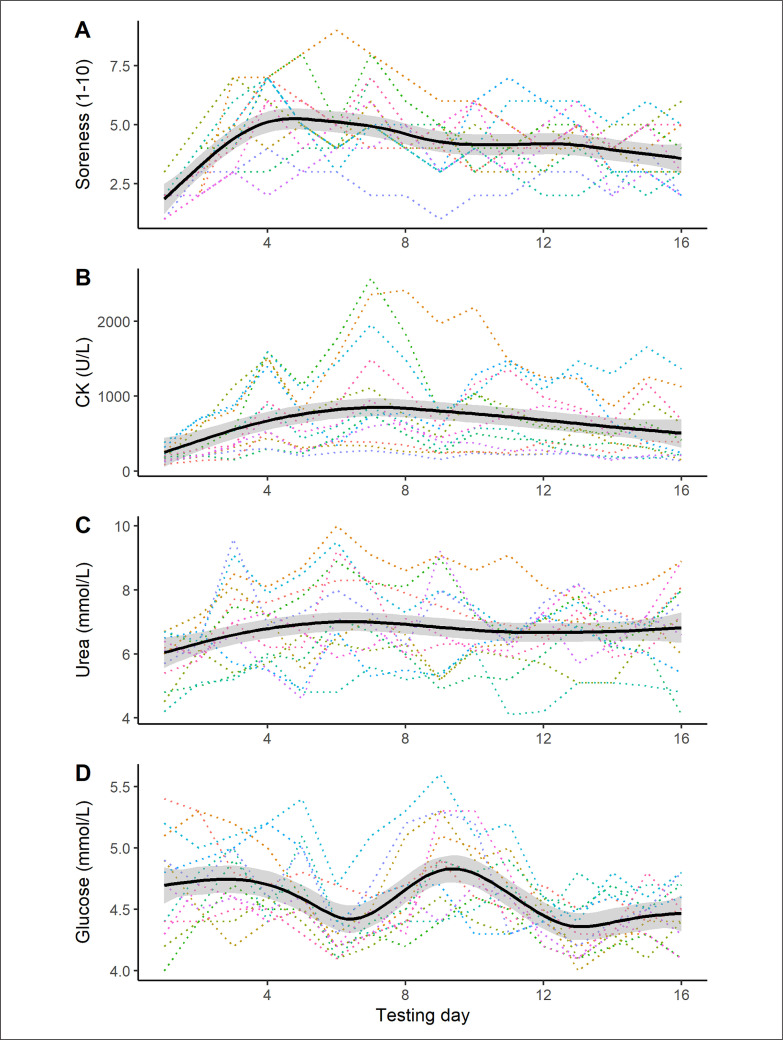
Time-series plots for the muscle damage and energetic variables in male boxers. The solid black line represents the smoothed trajectory, with the shaded region indicating the 95% CI. The dotted lines represent the individual time series. Key: CK = creatine kinase.

Regarding the group response, muscle soreness rose immediately after study inception, peaking on days 4–5 before declining slowly over the remaining days (Figure 1A). For plasma CK (Figure 1B), we saw a more gradual concentration increase up to day 7, followed by a slow decline over time. A relatively flat trajectory was observed for plasma urea concentration (Figure 1C), whereas plasma glucose activity (Figure 1D) was undulating over the 16-day period. Importantly, all variable measurements were characterized by large individual variability. T-test analyses (day 1 vs. day 16) revealed a non-significant decrease in body size (-0.4%, *p* = 0.118), but a significant increase in creatinine concentration (3.8%, *p* = 0.040).

**FIG. 2:**
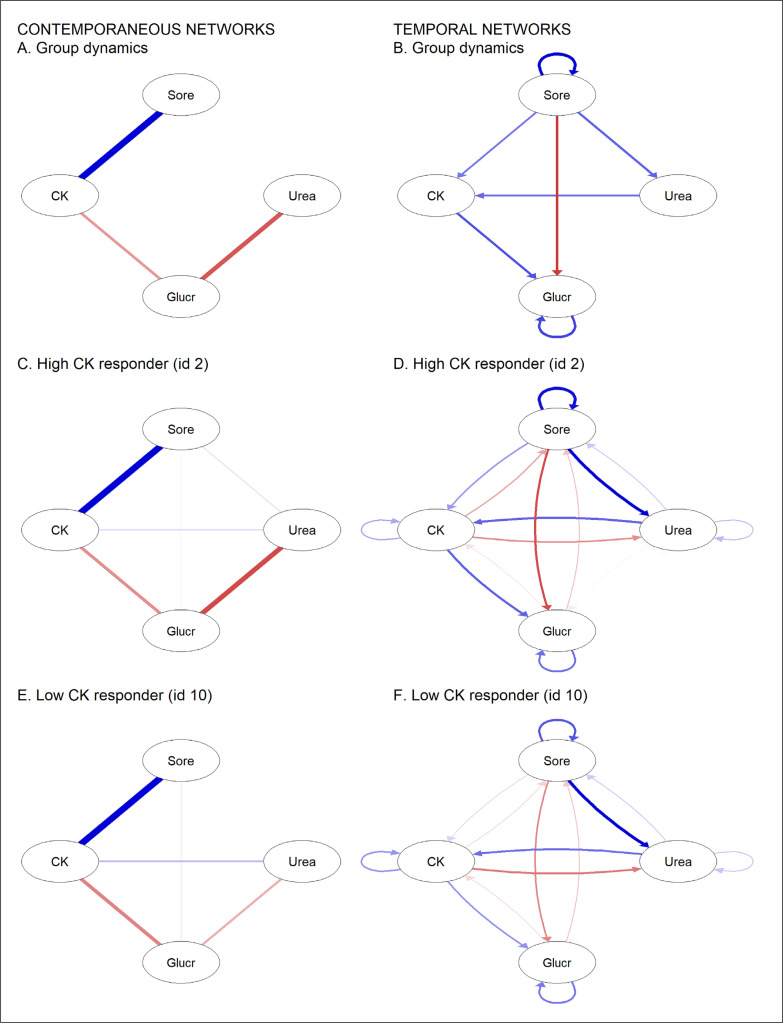
Within-person contemporaneous and temporal networks between the muscle damage and energetic variables in male boxers. Blue edges represent positive correlations and red edges indicate negative correlations. The mlVAR software hides non-significant edges in the group network, but cannot hide the same edges in subject networks. Key: CK = creatine kinase, Glucr = glucose residuals, Sore = muscle soreness.

Three significant edges emerged in the within-person contemporaneous network (Figure 2A), between the soreness and CK (*r* = 0.44), CK and glucose (*r* = -0.18), and glucose and urea nodes (*r* = -0.29). More significant connections were identified in the within-person temporal network (Figure 2B), with soreness exhibiting positive edges with CK (r = 0.19) and urea (*r* = 0.22), and a negative edge with glucose (*r* = -0.28). A positive urea and CK edge also emerged (*r* = 0.28) and the negative CK-glucose edge in the contemporaneous network was now positive (*r* = 0.25). To establish whether individuals differed in system dynamics, we explored the corresponding networks for boxers displaying the highest CK (id 2, Figure 2C and Figure 2D) and lowest CK means (id 10, Figure 2E and Figure 2F). For both participants, the contemporaneous and temporal networks were largely similar to the group response and each other.

The mlVAR package [19] can disaggregate time-series data to additionally estimate a between-person network, although the edges between nodes are biased when N is low. Consequently, we have not presented the between-person network in this study.

## DISCUSSION

This study sought to disentangle the day-to-day dynamics between muscle damage and energetics in male boxers within an ecological training environment. As a group, the muscle soreness, CK, urea, and glucose trajectories revealed different training patterns, but with a large degree of heterogeneity that arguably reflects within-person variances (from 47% up to 78%) from the mean. Within-person network analyses revealed some interrelatedness between these out-comes in the contemporaneous network, with more (but not always consistent) edges emerging in the temporal network.

Across different sports and levels of eliteness, initial return to structured training after the off-season period results in elevated concentrations of EIMD biomarkers [25]. The muscle soreness and CK profiles of our boxing sample concur with this evidence, increasing over the first few days of early-season training and remaining some-what elevated thereafter. Among boxing populations, the induction of EIMD is likely due to mechanical, metabolic, and oxidative stressors arising from repeated and initially unaccustomed exercise, combined with the physical trauma of full-contact sparring, heavy bag work, and related boxing drills [4, 6, 9, 10]. Other potential moderators of EIMD include training intensity [9], the number and locality of punches received when competing (or sparring) [5], dietary in-take (e.g., soy protein) [7], and use of any post-sparring recovery intervention [8]. We further demonstrated a positive within-person edge between soreness and CK in both networks; a finding that supports evidence from untrained adults [11, 12, 17] and male boxers [7] that muscle soreness and CK concentration tend to rise and fall in parallel or with a short lag.

Other results generated by the network models were contradictory. In the temporal network, greater muscle soreness correlated with higher urea and lower glucose levels 1-day later, whereas the same edges were absent based on time-matched (contemporaneous) comparisons. Daily fluctuations in urea and glucose concentrations were also related in the latter, but not the former, network. Finally, a CK and glucose edge emerged in both networks, but was differently expressed in the contemporaneous (negative) and temporal (positive) models. The pleiotropic role of energy biomarkers in muscle contractions, glycemic control, the inflammatory insult, and muscle recovery, could explain this diversity. Hence, it is problematic to limit these results to the silo of EIMD, especially when athletes are performing multiple training sessions each day and week to achieve different physiological responses. Rather, they should be interpreted from the broader prism of training and recovery, with muscle damage as a secondary outcome. Adding to these difficulties, upstream signals like pro-inflammatory cytokines (e.g., IFNγ, TNF) and glucocorticoids (e.g., cortisol) can affect glucose homeostasis [26, 27], and notwith-standing the direct effect of dietary intake on energetic biomarkers like glucose.

Our data indicate a high degree of individual variability in muscle damage and energetics, as seen in boxers [4, 5, 9], rugby players [28, 29], and untrained adults [11, 12, 17]. Physical impacts in a sporting contest can affect CK release [28, 29] and thus, drive this individuality, but sparring impacts from boxing were not measured. Others have highlighted the role of genetic factors. As an example, the highly variable CK response to exercise was attributed to polymorphisms in genes that encode proteins involved in CK release [11]. On the other hand, the network profiles of the highest CK (mean 1353 U/L) and lowest CK (mean 212 U/L) responder indicate similar dynamical interplay. This raises the intriguing possibility of adaptive mechanisms that synergize the training response to compensate for differences in baseline and reactive physiology, or it could reflect a predisposition that favors natural selection to the inherent demands of boxing. To test the uniformity and strength of these networks, one could take a case-study approach over several weeks and months of data collection [23, 30], thereby ensuring a more detailed and personalized inspection of EIMD to address questions around different training phase and/or workload effects [25].

On a practical level, the selected EMID and energetic biomarkers appear to be indicative of training responsivity and/or functional connectivity and thus, should form part of a standing testing battery in boxers. Researchers, coaches, and practitioners could also benefit from the creation of within-person networks, either for a training squad or individual athletes. One could explore the structure of a multivariate dataset, as we have, with network representations helping to communicate intricate patterns of interrelatedness [20]. For instance, we identified more edges in the temporal network, which suggests that a daily testing schedule and investigation of lagged effects can extract more meaningful results for boxers. Further possibilities exist to generate causal hypotheses for testing [20], such as our observation of coherent network patterns (across all boxers and high and low CK responders) reflecting training synergy or natural selection. The network approach further aligns to contemporary perspectives of sport processes that encompass complex systems principles (e.g., interdependence, temporal nestedness, circular causality) [23, 31, 32]. This conceptual shift from traditional models (e.g., univariate and non-lagged analyses, pre and post design) can better capture when, how, and why, different biomarkers interact in a sports environment, allowing a more targeted approach to athlete training, assessment and evaluation.

Caution should still be exercised when interpreting the current findings. Only a limited number of variables were collected and we did not quantify the training stressors, in particular sparring impact, each day. Moreover, the networks were estimated from sparse sampling and we fixed the temporal network at lag-1, which precludes detection of correlations that exist within, or beyond, the 1-day sam-pling interval. As a delimitation, any inferences are specific to male boxers of a similar age and amateur status, as well as the specific phase of training. The lack of a body-fat assessment is another limitation. Rapid weight loss can also affect CK and other energetic bio-markers in combat-sport athletes [33]. However, this work was conducted early in the boxing season and weight cutting for competition was not a goal, and prior weight cutting (> 1 month earlier) was not deemed relevant. Future work on boxers would benefit from study replication over a longer time period, with added biochemical (e.g., cytokines, neutrophils), endocrine (e.g., cortisol, testosterone) and/ or performance (e.g., muscle power, fatigue) measures [3], along-side a detailed anthropometric assessment (via bioimpedance or dual x-ray absorptiometry) to determine how changes in body composition affect blood biochemistry. Combining this information with daily exercising loads, including sparring impacts and their location (e.g., head vs. body), would help construct a more comprehensive ontology of training, EIMD, and recovery processes.

## CONCLUSIONS

To summarize, day-to-day fluctuations in muscle damage and energetic activity, which occurred in a normal physiological range among male boxers, were found to be highly variable during early-season training. Within-person network analyses identified some interrelatedness between the study outcomes, although the strength, direction, and even presence, of these relationships were contingent on temporal (lag-0 vs. lag-1) ordering. We attribute these inconsistences to the pleiotropy of energy biomarkers in training and recovery.
